# Tetanus seroprotection among children in the Democratic Republic of the Congo, 2013–2014

**DOI:** 10.1371/journal.pone.0268703

**Published:** 2022-05-19

**Authors:** Alvan Cheng, Angie Ghanem-Uzqueda, Nicole A. Hoff, Hayley Ashbaugh, Reena H. Doshi, Patrick Mukadi, Roger Budd, Stephen G. Higgins, Christina Randall, Sue Gerber, Michel Kabamba, Guilluame Ngoie Mwamba, Emile Okitolonda-Wemakoy, Jean Jacques Muyembe-Tanfum, Anne W. Rimoin

**Affiliations:** 1 Department of Epidemiology, Fielding School of Public Health, University of California, Los Angeles, California, United States of America; 2 School of Medicine, Kinshasa University, Kinshasa, Democratic Republic of the Congo; 3 DYNEX Technologies Incorporated, Chantilly, Virginia, United States of America; 4 Lentigen Technology, Incorporated, Gaithersburg, Maryland, United States of America; 5 Bill and Melinda Gates Foundation, Seattle, Washington, United States of America; 6 Expanded Program on Immunization, Ministry of Public Health, Kinshasa, Democratic Republic of the Congo; 7 VillageReach, Kinshasa, Democratic Republic of the Congo; 8 Kinshasa School of Public Health, Kinshasa, Democratic Republic of the Congo; 9 National Institute for Biomedical Research, Kinshasa, Democratic Republic of the Congo; Public Health England, UNITED KINGDOM

## Abstract

**Background:**

Tetanus is a potentially fatal disease that is preventable through vaccination. While the Democratic Republic of the Congo (DRC) has continued to improve implementing routine vaccination activities throughout the country, they have struggled to maintain high childhood vaccine coverage. This study aims to examine the seroprevalence of tetanus in children 6 to 59 months to identify areas for intervention and improvement of vaccination coverage.

**Methods:**

In collaboration with the 2013–2014 Demographic and Health Survey, we assessed the seroprevalence of tetanus antibodies among children in the DRC. Dried blood spot samples collected from children 6–59 months of age were processed using a prototype DYNEX Multiplier® chemiluminescent automated immunoassay instrument with a multiplex measles, mumps, rubella, varicella and tetanus assay. Multivariable logistic regression was used to determine factors associated with tetanus vaccination and seroprotection.

**Results:**

Overall, 36.1% of children 6–59 months of age reported receiving at least 1 dose of tetanus vaccine while 28.7% reported receiving 3 doses; tetanus seroprotection was 40%. Increasing age in children was associated with decreased tetanus seroprotection, but increased number tetanus vaccinations received. Factors related to increased tetanus seroprotection included number of children in the household, wealth index of the family, urban residence compared to rural, level of maternal education, and province and geography.

**Conclusions:**

Our findings in this nationally representative sample indicate that serology biomarkers may help identify children who are not fully immunized to tetanus more accurately than reported vaccination. While children may be captured for routine immunization activities, as children age, decreasing seroprevalence may indicate additional need to bolster routine vaccination activities and documentation of vaccination in school aged children. Additionally, the study highlights gaps in rural residential areas and vaccination coverage based on maternal education, indicating that policies targeting maternal education and awareness could improve the coverage and seroprevalence of tetanus antibodies in the DRC.

## Introduction

Tetanus is an infectious, non-communicable disease caused by exposure to the spores of the *Clostridium tetani* bacterium that affects the nervous system. This bacterium is found in the soil and intestinal tracts of animals and is ubiquitous, causing infections worldwide. Clinical disease is caused by neurotoxins produced under anaerobic conditions in wounds contaminated with the bacterial spores and characterized by muscle spasms [1, 2]. Tetanus infections are especially common and serious among neonates and their mothers when the mother is unprotected from infection during birth. The average case fatality rate can vary from 10% to 70% depending on treatment, age, general health of the patient, and quality and availability of hospital care. Fatality is almost 100% among the youngest and oldest patients in the absence of adequate care for both neonatal and non-neonatal tetanus [3–5]. Despite an overall decrease in tetanus related morbidity and mortality worldwide, tetanus remains endemic in resource limited settings with approximately 56,743 deaths due to tetanus in 2015; 19,937 in neonates and 36,806 in older children and adults. Of these deaths, 44% and 36%, respectively, occurred in sub-Saharan Africa [6]. In 2019, the Global Burden of Disease study estimated over 73,000 total tetanus cases, in which over 27,000 were neonatal tetanus infections [7].

Vaccination with a tetanus-toxoid containing vaccine is the only means of achieving immunity to tetanus (individuals who recover from tetanus infection do not receive natural immunity and can be re-infected) and the initial three doses during routine immunization activities provide the foundation for building lifelong immunity to tetanus [8]. Initial immunity wanes after each dose of vaccine, but after three doses, immunity levels presumed to be protective are induced, and protection typically persists for 3–5 years, though immunity does begin to wane after the third dose. Following completion of the primary series of three doses, subsequent booster doses can further prolong immunity into adolescence and potentially much of adulthood if ≥5 doses are received according to the recommended schedule [8, 9].

Monitoring vaccination coverage is a critical element of assessing vaccination program effectiveness and ensuring population protection from vaccine preventable diseases (VPDs). Published reports typically rely on vaccination records, that may be unavailable or incomplete, national household surveys, such as the Demographic Health Survey (DHS), or parental recall. However, these sources of information may not be reliable. Maternal recall of vaccination is known to be subject to information bias, especially for older children [10], and all sources of household vaccination information (including vaccination cards) tended to misestimate coverage when compared to information obtained from a medical provider [11]. In low- to middle-income countries, evidence suggests that officially reported vaccination coverage estimates may require validation to accurately evaluate vaccination programs and vaccination coverage changes over time [12–14]. Serosurveys performed in conjunction with coverage surveys can provide more complete information about who is protected against VPDs and may provide a more accurate representation of vaccination coverage [3, 8, 15–17]. However, additional studies are needed to further evaluate serology compared to household vaccination information [18].

The Democratic Republic of the Congo (DRC) is the largest country in sub-Saharan Africa with an estimated population of 86.8 million inhabitants [19]. The majority of the population continues to live in rural and often times very isolated communities that are extremely difficult to access due to poor road infrastructure. In the most recent estimates, DRC was ranked 12th for highest infant mortality rates [20]. The DRC includes tetanus vaccination in the routine childhood immunization schedule through use of the pentavalent Diphtheria, Tetanus, Pertussis, *Haemophilus influenzae* and Hepatitis B vaccine given at 6 weeks, 10 weeks, and 14 weeks, following WHO recommendations for an accelerated schedule of three infant doses with at least 4 weeks between doses to be completed by 6 months of age [9], but does not currently provide the booster doses as part of the national vaccination schedule. In 2013, vaccination coverage for the first and third doses of tetanus vaccine in the DRC were estimated to be 74% and 68%, respectively, among children 12–23 months of age; however, these estimates from WHO and UNICEF are based on information of varying quality which may not be reliable [21].

Ensuring adequate immunity among children in the DRC through widespread vaccination coverage is the first step to eliminating tetanus infections among children and adults. This study aims to assess the seroprevalence of tetanus antibodies and establish demographic predictors of seroprotection among children in the DRC, in order to identify potential areas of intervention for improving vaccine coverage.

## Methods

### Study population and design

The second DHS (EDS-RDC II) conducted in the DRC took place from November 2013 to February 2014 using a 2-staged stratified cluster design [22]. These surveys provide nationally representative estimates on maternal and child health, as well as basic demographic and health information [23]. Details on the sampling design and data collection procedures are described elsewhere [24]. Data were collected from a nationally representative sample of 18,171 households, survey data on children 6 to 59 months of age were collected for all households where the biological mother was present for an interview. In addition to survey data, biological specimens in the form of dried blood spots (DBS) were collected from children after parental consent, in households from which men were selected to participate in an individual interview (approximately 50% of the total selected households) [22, 24, 25].

All survey data from the paper questionnaires were converted to an electronic format using the Census and Survey Processing System (US Census Bureau, ICF Macro, Rockville, MD). All questionnaires are double entered and checked for inconsistencies by comparing the two datasets. DBS samples were analyzed for biomarker data to assess population immunity to select VPDs.

### Laboratory analyses

DBS samples were extracted using a modified extraction protocol [26] and processed at the UCLA-DRC laboratory in collaboration with National Laboratory for Biomedical Research (INRB) in Kinshasa, DRC. Briefly, a 0.25" DBS punch was extracted, shaken at room temperature in 1ml phosphate buffered saline, 0.05% Polysorbate 20, and 5% dried milk, which represents a 1:143-fold dilution assuming 7μl of serum per punch. The DYNEX Multiplier® chemiluminescent automated immunoassay platform with a research use-only kit for measles, mumps, rubella, varicella-zoster virus, and tetanus (MMRVT) was used to test samples for IgG antibody response. Polystyrene beads coated separately with antigen to measles, mumps, rubella, varicella-zoster, and tetanus were immobilized within 54-well Multiplier® assay strips with 10 beads per well. An assay score (AS) for each sample was calculated as a ratio of the signal to a five-donor, pooled positive control (PPC) included in each run. Based on validation and epidemiologic studies, the positive/negative cutoff for tetanus IgG antibody detection was set at an AS of 0.069 (corresponding to 200 mIU/ml), which is considered an appropriate seroprotective cutoff when determined via ELISA [8, 27].

### Statistical analyses

Using the EDS-RDC II data, we assessed the seroprevalence of tetanus antibodies among children 6 to 59 months in the DRC. χ^2^ analyses were conducted on the weighted samples to assess sociodemographic differences between serologic test results (positive and negative for tetanus antibodies). Independent predictors of seroprotection were identified through bivariate weighted logistic regression models. Vaccination was not included in our model of predictors as it likely mediates many of these associations. Weighted multivariable logistic regression models were initially run with all variables; only significant predictors based on backwards selection using Bayesian information criterion (BIC) were retained in the final model stratified by residence type (urban vs. rural) [28, 29]. Maps of tetanus seroprotection by province were created to examine the spatial distribution of serologic response in children. In sub-analyses, we utilized serology biomarkers to assess the accuracy of reported vaccination information collected through DHS, by evaluating the number of pentavalent vaccine doses received against tetanus serology as the gold standard. Data on the number of vaccine doses are obtained from maternal recall and vaccination card.

All analyses accounted for the cluster survey design and were conducted using SAS software, version 9.4 (SAS Institute, Cary, NC), and maps were created using ArcGIS software version 10.6 (ESRI, Redlands, CA).

### Ethical approval

Ethical approval was obtained at UCLA Fielding School of Public Health, the Kinshasa School of Public Health, and the Centers for Disease Control and Prevention. Informed consent was obtained from all enrolled participants as a part of the DHS survey. Before each interview or biomarker test is conducted, an informed consent statement is read to the respondent; a parent or guardian must provide consent prior to participation by a child or adolescent. Written informed consent was taken from the head of the households and study participants before data collection; written informed consent was obtained from mothers before their children were allowed to participate.

## Results

**Fig 1:**
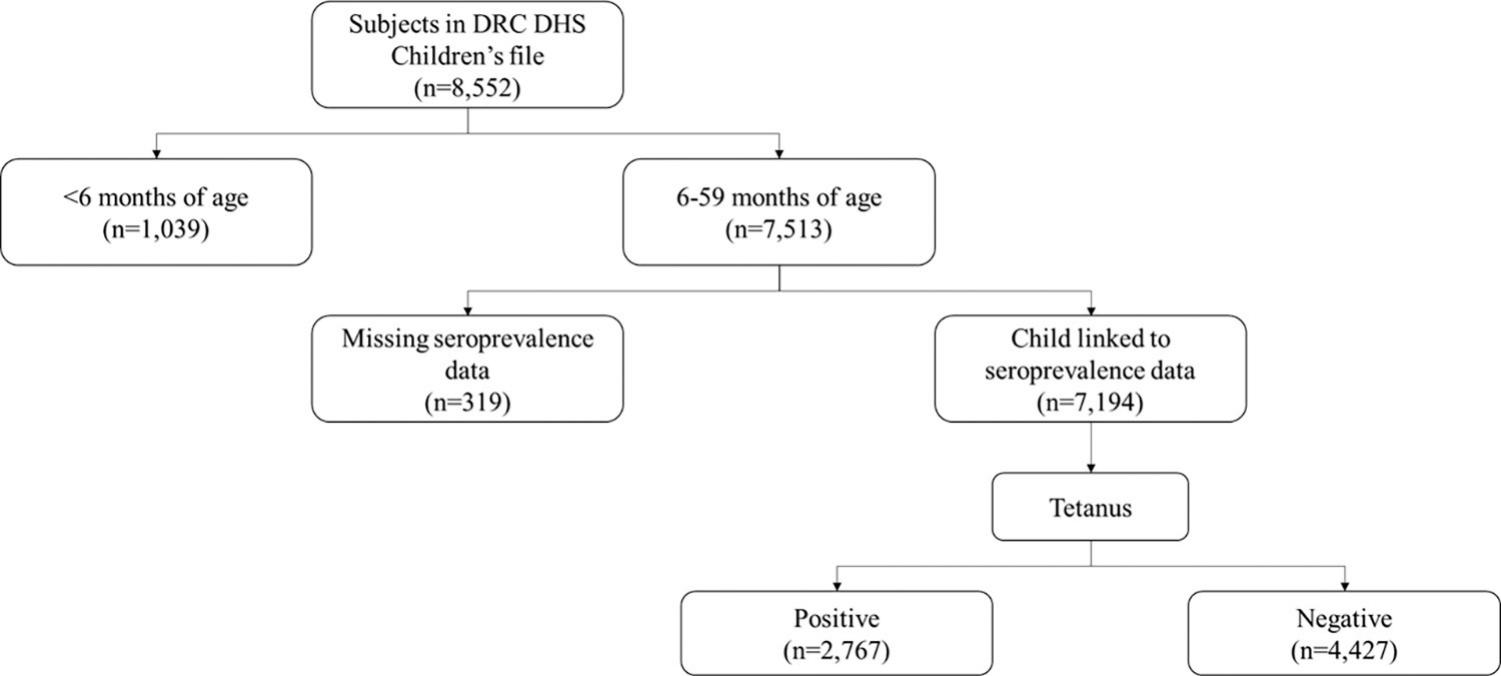
Attrition table. Study inclusion for assessment of tetanus seroprevalence among 6- to 59-month-old EDS-RDC II respondents (unweighted).

In total, 7,194 children between 6 and 59 months of age had tetanus antibody results, representing a weighted sample of 7,249 children; 3,634 male (50.1%) and 3,615 female (49.9%). Overall, 40.4% (n = 2,930) of children were seropositive for tetanus antibodies and 59.6% (n = 4,319) were seronegative. Of the total DHS sample, 319 children were missing results and excluded from the analyses (Fig 1).

**Fig 2:**
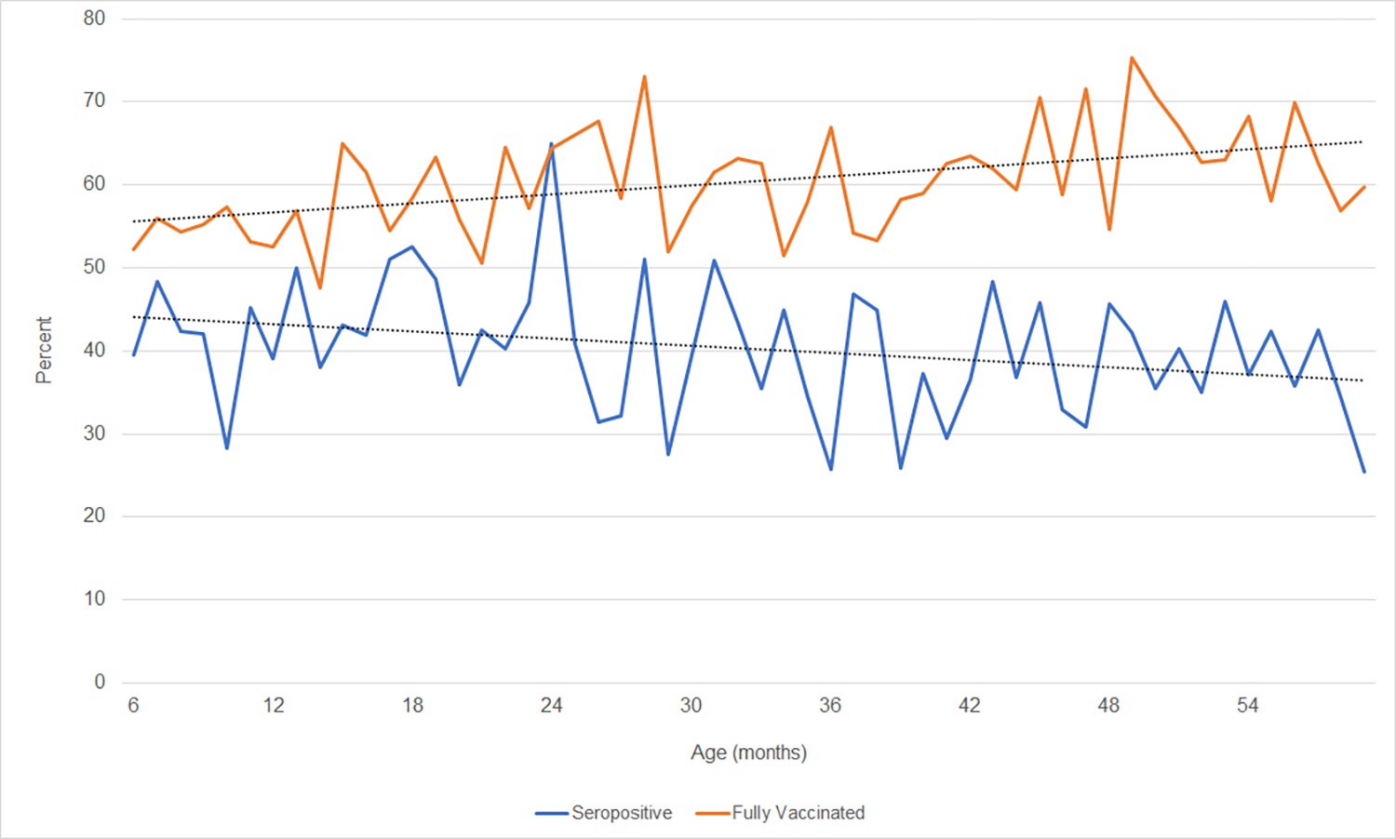
Tetanus seropositivity and vaccination trends by age. Tetanus seroprotection and full vaccination coverage (documentation of three doses of tetanus containing vaccine) according to age (in months) among children 6–59 months old in the EDS-RDC II (linear trend lines in black).

Tetanus seroprotection differed between males (38.6%) and females (42.3%) (Table 1). Percent seroprotection decreased with increasing age, from 40.8% of 6- to 11-month-olds testing positive to 38.8% of 4-year-olds. However, the percent of reported full vaccination displayed a positive trend with age, from 55.0% of 6- to 11-month-olds to 64.2% of 4-year-olds reporting full vaccination (Fig 2); the percent of reported full vaccination among all children 6–59 months was 60.2% (S1 Table). Percent seroprotection decreased with increasing number of children in the household and was higher among firstborn children. Percent seroprotection increased with higher level of maternal education, greater wealth index of the family, and was higher in urban areas versus rural areas (48.0% versus 37.2%, respectively) (Table 1).

**Table 1:** Weighted demographic characteristics by tetanus serostatus result of children 6–59 months old in the EDS-RDC II.

	Tetanus serostatus		
Characteristic	Positive	Negative	Chi-square p-value
	(n = 2930)	(n = 4319)	
	n (%)	n (%)	
Child’s age category^a^			0.0254
6–11 months	347 (40.8)	503 (59.2)	
1 year	748 (44.2)	943 (55.8)	
2 years	683 (41.1)	978 (58.9)	
3 years	570 (36.9)	977 (63.1)	
4 years	582 (38.8)	919 (61.2)	
Child’s sex			0.0406
Male	1401 (38.6)	2233 (61.5)	
Female	1529 (42.3)	2086 (57.7)	
No. of children in household^b^			< .0001
1	533 (50.3)	526 (49.7)	
2	592 (40.2)	879 (59.8)	
3	574 (41.4)	811 (58.6)	
4	397 (33.0)	807 (67.1)	
≥5	835 (39.2)	1296 (60.8)	
Birth order^c^			< .0001
Firstborn	643 (48.1)	693 (51.9)	
Non-firstborn	2287 (38.7)	3626 (61.3)	
Mother’s age at child’s birth (years)			0.1967
≤20	541 (44.2)	684 (55.8)	
21–25	793 (40.4)	1170 (59.6)	
26–30	767 (40.5)	1125 (59.5)	
31–35	451 (37.6)	749 (62.4)	
36+	378 (39.0)	591 (61.0)	
Mother’s highest education			< .0001
None	606 (41.4)	856 (58.6)	
Primary	1113 (35.2)	2046 (64.8)	
Secondary/higher	1211 (46.1)	1417 (53.9)	
Wealth index^d^			< .0001
Poorest	556 (34.1)	1076 (65.9)	
Poorer	603 (35.7)	1088 (64.3)	
Middle	597 (40.6)	873 (59.4)	
Richer	588 (43.4)	767 (56.6)	
Richest	587 (53.3)	515 (46.8)	
Old Province			< .0001
Bandundu	433 (35.2)	798 (64.8)	
Bas-Congo	129 (40.9)	187 (59.1)	
Equateur	401 (37.2)	678 (62.8)	
Kasai-Occidental	209 (37.2)	353 (62.8)	
Kasai-Oriental	314 (39.4)	482 (60.6)	
Katanga	240 (32.1)	508 (67.9)	
Kinshasa	275 (57.3)	205 (42.7)	
Maniema	88 (34.5)	167 (65.5)	
Nord-Kivu	330 (53.9)	282 (46.1)	
Orientale	268 (43.1)	354 (56.9)	
Sud-Kivu	243 (44.2)	307 (55.8)	
Residence			< .0001
Urban	1039 (48.0)	1127 (52.0)	
Rural	1891 (37.2)	3192 (62.8)	
Reported number of tetanus vaccine doses^e^			< .0001
0	323 (22.2)	1133 (77.8)	
1	167 (32.6)	345 (67.4)	
2	360 (39.8)	543 (60.2)	
3	2073 (47.8)	2268 (52.3)	

^a^Only children 6 months of age and older were invited to participate in the serosurvey.

^b^Children in household is the sum of boys and girls that currently live in the household.

^c^Birth order ranges from firstborn to 15th-born.

^d^Wealth index is a composite measure of a household’s cumulative living standard based on household ownership of selected assets, materials used for housing construction, and types of water access and sanitation facilities. Using principal components analysis, the DHS separates all interviewed households into 5 wealth quintiles.

^e^Reported number of vaccine doses is the sum of vaccine doses received reported by either maternal recall or vaccination card.

**Fig 3:**
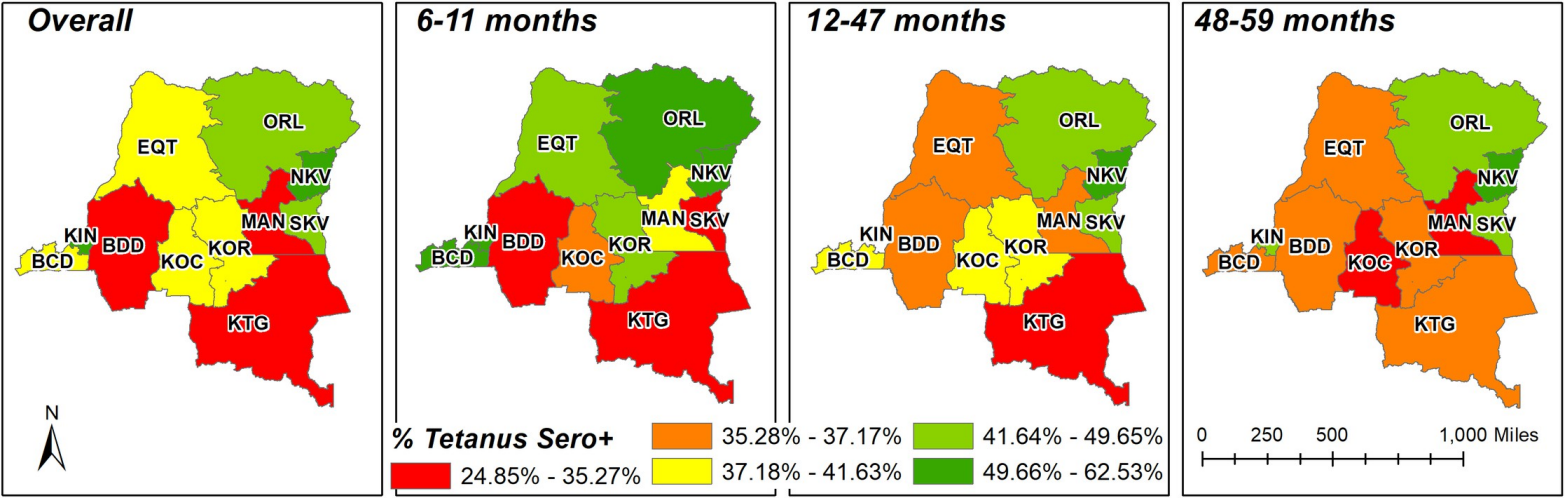
Tetanus seroprotection by province and age among children 6–59 months old in the EDS-RDC II.

There were differences in the percent of children who were seroprotected among the provinces. The lowest was observed in Katanga (32.1%) and Maniema (34.5%) and highest in Kinshasa (57.3%), and Nord-Kivu (53.9%) (Table 1). Examining the geographic distribution of seroprotection by age group shows additional variation by province. Seroprevalence of tetanus antibodies remained high in Kinshasa in each age group except for the 48- to 59-month-old children, where it dropped to 43.1%. Seroprevalence was highest in Nord-Kivu among 6- to 11-month-old children (61.2%) and in Kinshasa among 12- to 47-month-old children (62.5%) (Fig 3).

**Fig 4:**
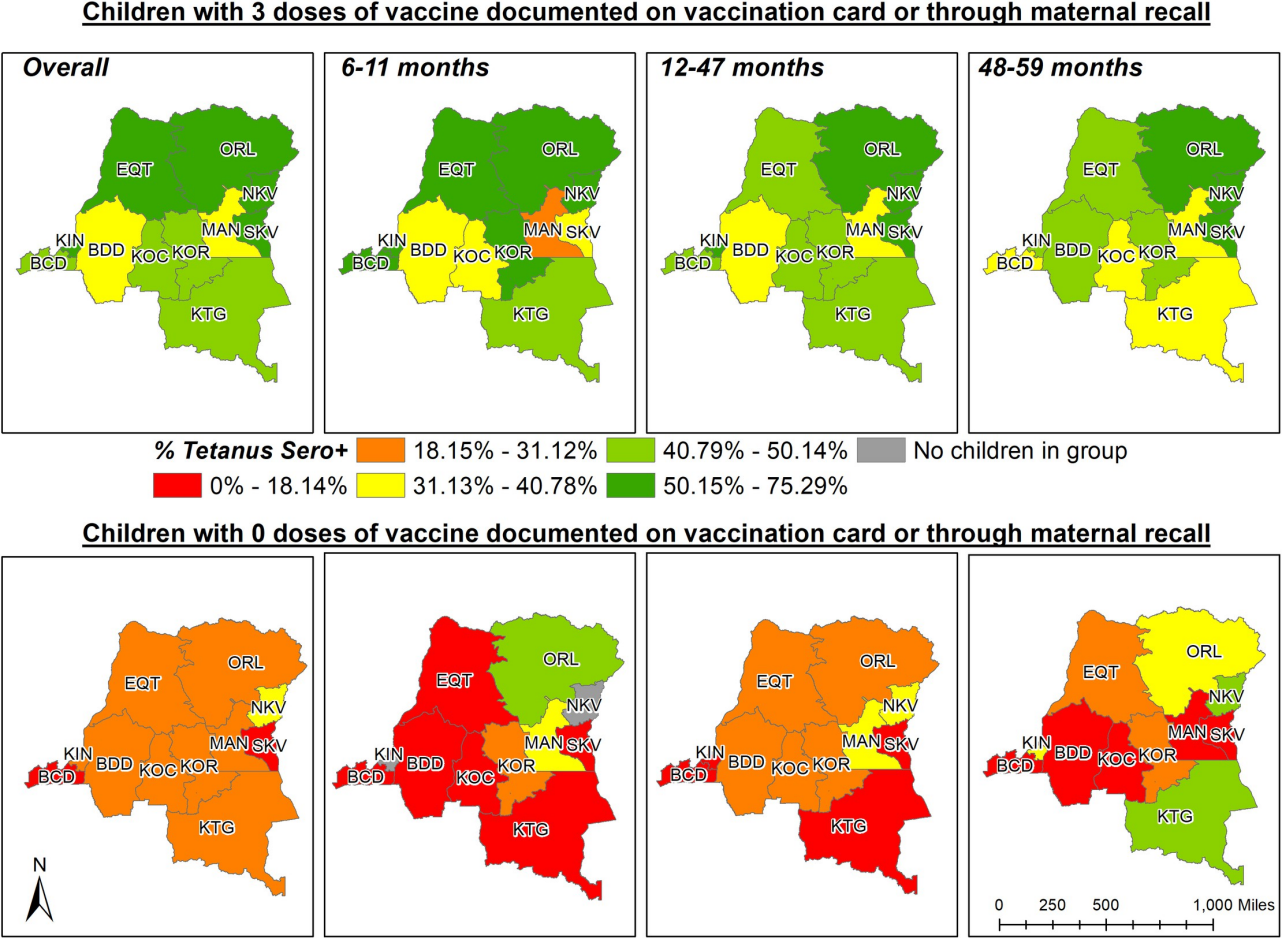
Tetanus seroprotection by province and age among children 6–59 months old who reported receiving 0 vs. 3 doses of tetanus containing vaccine in the EDS-RDC II.

Overall, tetanus seroprotection was low among children in the DRC, regardless of the number of tetanus vaccine doses reported, but increased from 32.6% to 47.8% among those receiving one versus three doses of tetanus vaccine, respectively. However, 22.2% of children who reported zero doses of tetanus vaccine also demonstrated tetanus seroprotection and this was seen across all provinces (Table 1). While tetanus seroprotection among children reported as receiving three doses was generally higher than among children with zero doses, there was still evidence of tetanus seroprotection among children with zero documented doses in all provinces. The largest discordance between serology and vaccination record was observed in the oldest age group, 48–59 months, and in the southeastern provinces of the DRC (Fig 4).

**Fig 5:**
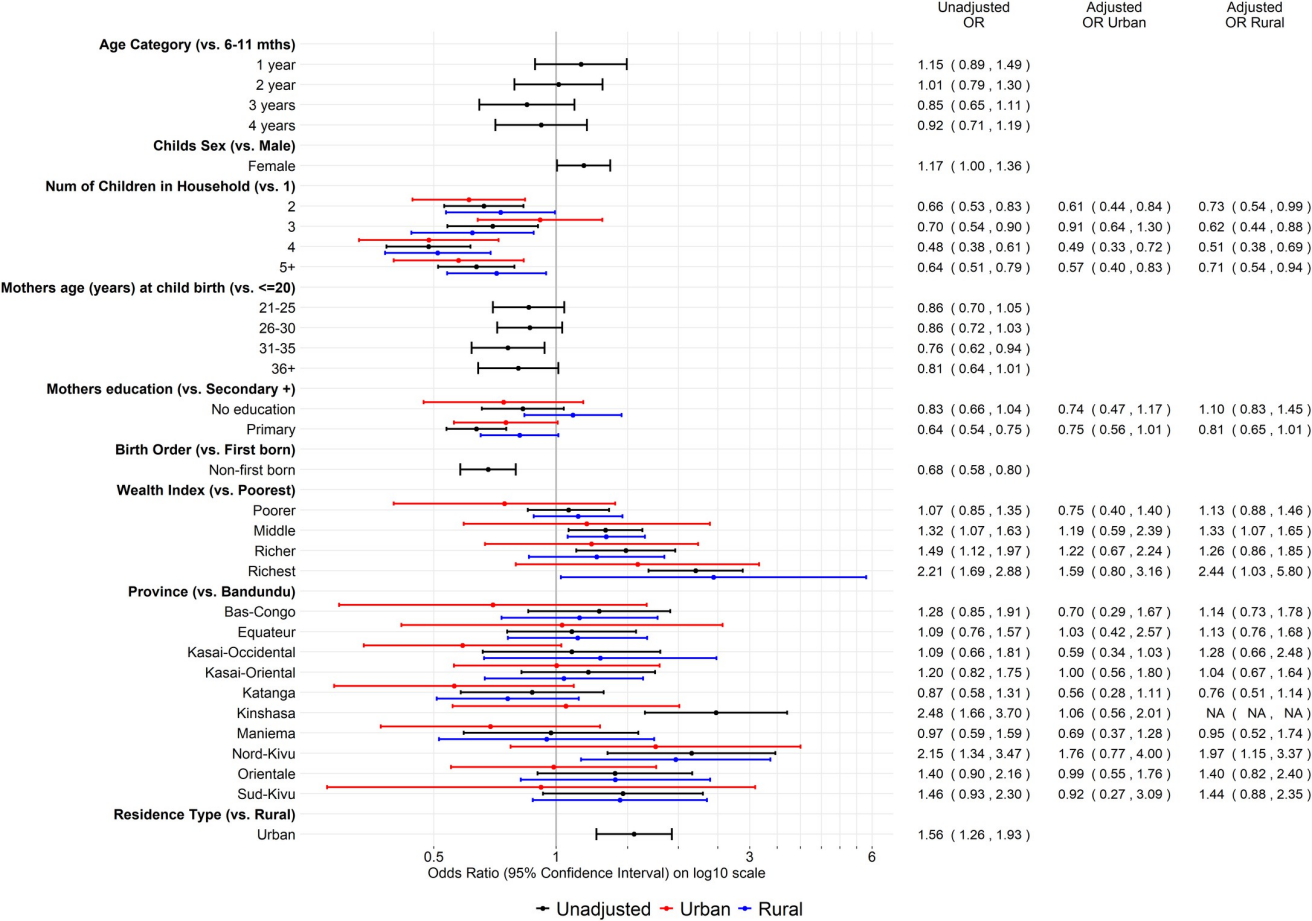
Odds ratios of tetanus seroprotection. Forest plot of odds ratios from weighted logistic regression of sociodemographic factors/characteristics associated with tetanus seroprotection among children 6–59 months old in the EDS-RDC II, unadjusted and stratified by residence type (adjusted for all other variables in the model).

In the multivariable analysis among urban residences, an increased number of children in the household was associated with a lower odds of seroprotection compared to only one child (Fig 5). The odds of seroprotection were lower among children whose mother had less than a secondary education and were lower among children in Katanga, Kasai-Occidental, Maniema, and Bas-Congo compared to in Bandundu. Similarly, in the multivariable analysis among rural residences, the odds of seroprotection were lower among those with more than one child in the household compared to only one child. The odds of seroprotection were positively associated with wealth index, specifically in the middle and richest areas compared to poorest, and were higher in most provinces compared to Bandundu, especially Nord-Kivu, but was lower among children in Katanga (Fig 5).

## Discussion

In this nationally representative sample of children 6–59 months, our results indicate that seroprotection against tetanus is low among children throughout the DRC and that there are discrepancies between serology results and documentation of tetanus vaccination. We found that the seroprevalence of tetanus-specific IgG antibodies decreased with increasing age and number of children in the household, was positively associated with increased maternal education level and increased wealth index, and varied by province and residence type.

Although serological biomarkers are unable to indicate the number of vaccine doses received [30], the decrease in tetanus seroprotection among older children in this study may suggest that children are not receiving the complete series, despite information gathered and documented in DHS from the vaccination record. Among children 6–11 months, 12–47 months, and 48–59 months of age who reported receiving three doses, only 50.6%, 48.3%, and 44.6% demonstrated protective immune response respectively (S1 Table). While antibody levels typically decrease slowly after each initial dose of tetanus-containing vaccine, the immune response to receiving all three doses is typically robust and should provide protective levels of immunity for children for 3–5 years [8, 9]. Conversely, among children 6–11 months, 12–47 months, and 48–59 months of age who reported receiving no vaccination through routine immunization, 16.9%, 21.9%, and 27.0% demonstrated immunity to tetanus respectively (S1 Table). As children who received no vaccination should not test positive for tetanus IgG antibodies, our results indicate that reported vaccination, from either vaccination cards or by the mother, may not be a reliable indicator of vaccine coverage and is likely subject to reporting errors [11, 15, 31]. Similar instances of tetanus seropositivity among those not considered protected based on vaccination history have been observed as well [16]. For diseases like tetanus, seroprevalence surveys might be a more accurate source of vaccination information when there are doubts about vaccination card reliability or validity of maternal recall and should also be used to ascertain information about appropriate seroconversion among children, though the relationship between tetanus vaccine coverage and seroprevalence needs to be explored further. Furthermore, relying only on maternal recall or even vaccination card information may lead to targeting groups that are already vaccinated, resulting in resource misuse while leaving truly unvaccinated children unprotected from VPDs [10].

Our finding of decreasing tetanus seroprotection with older age among young children is consistent with other studies [32–35]. Studies in Kenya and Tanzania have observed tetanus immunity gaps among children 5–14 years of age [35]. In Kenya, tetanus seroprotection decreased from 90% among 1–4 year old children to 66% among 5–14 year old children. Similarly in Tanzania, compared to children 1–4 years of age, tetanus seroprotection among children 5–14 years of age decreased from 89% to 66% [35]. Although neither of these results are nationally representative, these results do reveal the potential for waning tetanus immunity among older children in a similar setting, potentially due to incomplete tetanus vaccination. Similarly, we noted lower odds of seroprotection with having more children in the household. In line with our findings, Akmatov and Mikolajczyk found that a higher number of children in households was associated with delayed and missing vaccinations [36], which should lead to lower levels of seroprotection.

The overall trend of increasing tetanus seroprotection with higher levels of maternal education may suggest that improving the educational standards of adolescent girls and women would result in increased childhood VPD immunity. Several studies have noted the association between maternal education and complete immunization among children, with some suggesting maternal literacy as a mediator in this pathway [37–40]. Regardless, it seems clear that female education is crucial for improving vaccination coverage among children. We also noted increased tetanus seroprotection with increased wealth index. In line with our findings and previous work [28], studies have found that wealthier families were more likely to fully vaccinate their children [41–47]. Acharya and colleagues showed that higher wealth status and mothers having secondary or higher level of education were positively correlated with full childhood immunization coverage in the DRC, likely due to better access and use of health services [41, 48].

Similar to previous findings on vaccine coverage [28], we also noted that geographic location and residence type were associated with tetanus serostatus. Residents in urban areas were more likely to be seropositive compared to their rural counterparts [47, 49]. This may be due to higher travel costs and longer distances to health facilities or poorer vaccine storage and handling in rural areas. The highest tetanus seroprevalence was found in Kinshasa and Nord-Kivu, while the lowest were in Maniema and Katanga. Kinshasa is the capital and Nord-Kivu is an area with ongoing conflict and humanitarian crisis. Emergency vaccination campaigns have been used in this area, especially among internally displaced people, which may contribute to the higher seroprevalence in this province [50]. However, the underlying reasons for varying seroprevalence across provinces remain unclear.

We were limited by the information gathered in the DHS survey to describe and elucidate tetanus seroprotection in DRC. Discrepancies observed between serology and reported vaccination may be a result of either failure to generate a protective IgG response, errors in reporting/documentation, disruptions in vaccine cold chain, suboptimal timing between doses, or supplemental immunization activities in the DRC; however, we do not have the information to examine this further. Additionally, information regarding booster doses was not ascertained in the DHS survey and could not be assessed in this study. Other limitations include potential misclassification of serostatus; however, comparison with commercially available kits and validation with confirmed samples tested on other multiplex panels, the multiplex assay exhibited high sensitivity and specificity for other infectious diseases including measles, mumps, rubella and varicella (sensitivity range: 89.5–100%; specificity range: 77.3–100%) [51]. Additionally, the chosen testing platform was highly cost-effective as we were able to evaluate the seroprevalence of several other vaccine-preventable diseases (mumps, measles, rubella, and varicella). Finally, while maternal antibodies are likely waning by 6 months of age [52], it is not possible to determine whether seroprotection in the youngest children was a result of vaccination or persistent maternal antibodies.

The DRC has taken important steps to ensure tetanus immunity among children by introducing the first three doses of tetanus containing vaccine. However, these nationally representative estimates demonstrate that overall vaccine-induced seroprotection to tetanus is low among children in the DRC, especially among older children, those in rural areas or in households with more than one child, and children whose mother has less than a secondary level of education. These trends can inform interventions for the Ministry of Health to improve children’s health and increase immunity to tetanus, such as including supplemental immunization activities (SIAs), targeting areas most at risk of low immunization coverage, or incorporating booster doses for older children. Frequent and regular SIAs could both increase the number of children receiving and completing the primary series of three doses and serve to provide the recommend booster doses, improving overall vaccine coverage and immunity against tetanus. Although children 6–59 months of age are only a part of the population at risk for non-neonatal tetanus, ensuring high tetanus vaccination rates plays a major role in preventing maternal and neonatal tetanus by prolonging protective immunity among adolescent females and women of reproductive age. This study adds to the literature examining the status of vaccine coverage and immunity to vaccine preventable disease in the DRC; however, additional serologic studies in older populations are necessary to improve tetanus elimination strategies, primarily for maternal and neonatal tetanus. This study also provides additional information about using serology biomarkers to describe vaccination history. Incorporating serology biomarkers can help identify the prevalence of protected individuals in addition to assessing vaccination coverage, offering an impression of public health risk. Understanding the current situation of tetanus seroprotection among children can inform future policy and public health interventions on improving tetanus immunity in the DRC.

## Supporting information

S1 Table Weighted demographic characteristics by reported tetanus vaccination of children 6–59 months old in the 2013–2014 DRC-DHS. (DOCX)
