# Supplementary material for: Tetanus seroprotection among children in the Democratic Republic of the Congo, 2013–2014
Source: PLoS One. 2022 May 19;17(5):e0268703. doi: 10.1371/journal.pone.0268703 (PMC9119496; doi:10.1371/journal.pone.0268703)
Supplement: S1 Table — (DOCX) [file pone.0268703.s001.docx]

**S1 Table.** Weighted demographic characteristics by reported tetanus vaccination of children 6–59 months old in the 2013–2014 DRC-DHS.

|  | Tetanus immunization | |  |
| --- | --- | --- | --- |
| Characteristic | Full | Partial/none | Chi-square p-value |
|  | (n=4341) | (n=2871) |  |
|  | n (%) | n (%) |  |
| Child’s age category^a^ |  |  | 0.0030 |
| 6-11 months | 466 (55.0) | 382 (45.0) |  |
| 1 year | 960 (56.9) | 726 (43.1) |  |
| 2 years | 1015 (61.5) | 636 (38.5) |  |
| 3 years | 940 (61.3) | 593 (38.7) |  |
| 4 years | 959 (64.2) | 534 (35.8) |  |
| Child’s sex |  |  | 0.8035 |
| Male | 2171 (60.0) | 1447 (40.0) |  |
| Female | 2170 (60.4) | 1423 (39.6) |  |
| No. of children in household^b^ |  |  | 0.0321 |
| 1 | 641 (60.8) | 414 (39.2) |  |
| 2 | 800 (54.5) | 667 (45.5) |  |
| 3 | 877 (63.8) | 498 (36.3) |  |
| 4 | 732 (61.1) | 465 (38.9) |  |
| ≥5 | 1292 (61.0) | 826 (39.0) |  |
| Birth order^c^ |  |  | 0.2914 |
| Firstborn | 826 (62.0) | 507 (38.0) |  |
| Non-firstborn | 3515 (59.8) | 2364 (40.2) |  |
| Mother’s age at child’s birth (years) |  |  | 0.2522 |
| ≤20 | 713 (58.4) | 508 (41.6) |  |
| 21-25 | 1187 (60.8) | 765 (39.2) |  |
| 26-30 | 1170 (62.2) | 710 (37.8) |  |
| 31-35 | 730 (61.0) | 466 (39.0) |  |
| 36+ | 542 (56.3) | 421 (43.8) |  |
| Mother’s highest education |  |  | <.0001 |
| None | 770 (53.1) | 680 (46.9) |  |
| Primary | 1688 (53.8) | 1453 (46.3) |  |
| Secondary/higher | 1883 (71.8) | 738 (28.2) |  |
| Wealth index^d^ |  |  | <.0001 |
| Poorest | 727 (44.9) | 892 (55.1) |  |
| Poorer | 865 (51.5) | 816 (48.5) |  |
| Middle | 837 (57.2) | 626 (42.8) |  |
| Richer | 992 (73.4) | 360 (26.6) |  |
| Richest | 920 (83.8) | 177 (16.2) |  |
| Old Province |  |  | <.0001 |
| Bandundu | 811 (66.2) | 415 (33.8) |  |
| Bas-Congo | 252 (80.6) | 61 (19.4) |  |
| Equateur | 414 (38.5) | 663 (61.5) |  |
| Kasai-Occidental | 319 (57.2) | 239 (42.8) |  |
| Kasai-Oriental | 405 (51.3) | 383 (48.7) |  |
| Katanga | 401 (53.8) | 345 (46.2) |  |
| Kinshasa | 412 (86.4) | 65 (13.7) |  |
| Maniema | 108 (42.7) | 145 (57.3) |  |
| Nord-Kivu | 525 (85.7) | 88 (14.3) |  |
| Orientale | 280 (45.7) | 333 (54.3) |  |
| Sud-Kivu | 415 (75.5) | 135 (24.5) |  |
| Residence |  |  | <.0001 |
| Urban | 1652 (76.7) | 503 (23.4) |  |
| Rural | 2690 (53.2) | 2368 (46.8) |  |
| ^a^Only children 6 months of age and older were invited to participate in the serosurvey.  ^b^Children in household is the sum of boys and girls that currently live in the household.  ^c^Birth order ranges from firstborn to 15th-born.  ^d^Wealth index is a composite measure of a household’s cumulative living standard based on household ownership of selected assets, materials used for housing construction, and types of water access and sanitation facilities. Using principal components analysis, the DHS separates all interviewed households into 5 wealth quintiles. | | | |
|  |  |  |  |
|  |  |  |  |
